# Seismic isolation retrofitting solution for an existing steel cable-stayed bridge

**DOI:** 10.1371/journal.pone.0200482

**Published:** 2018-07-30

**Authors:** Ahad Javanmardi, Zainah Ibrahim, Khaled Ghaedi, Niaz Bahadur Khan, Hamed Benisi Ghadim

**Affiliations:** 1 Civil Engineering Department, Faculty of Engineering, University of Malaya, Kuala Lumpur, Malaysia; 2 School of Mechanical and Manufacturing Engineering, National University of Sciences and Technology, Islamabad, Pakistan; 3 Sustainable and Innovative Bridge Engineering Research Center (SIBERC), Faculty of Civil Engineering, Fuzhou University, Fuzhou-Minhou, China; University of Pittsburgh, UNITED STATES

## Abstract

This paper investigated the seismic retrofitting of an existing cable-stayed bridge through the use of a seismic isolation system. The bridge is situated in a high seismic zone. During the Saguenay earthquake 1988, one of the anchorage plates of the bridge supports failed. Herein, several configurations of seismic isolation system were considered to identify an appropriate solution for the seismic retrofitting of the bridge in both the longitudinal and transverse directions. A three-dimensional model of the bridge was created, and its seismic behavior studied through nonlinear dynamic time-history analysis. The comparative performance study among the five retrofitting configurations showed that the partial seismic isolation of the bridge led to an enhancement of the seismic response of the bridge in one direction only. However, the overall seismic response of the cable-stayed bridge substantially improved in the longitudinal and transverse directions in cases where the isolation systems were utilized between the supports and the deck-tower connection of the bridge.

## Introduction

In recent decades, the construction of cable-stayed bridges has rapidly increased around the globe due to certain advantages, e.g., pleasing aesthetics, lighter weight, and longer span as a result of the usage of smaller structural members, and, hence, higher load capacity. Conversely, they are associated with a longer fundamental period, low structural damping, and greater flexibility, which makes them susceptible to large amplitude oscillation under earthquake excitations [1,2]. Hitherto, no approach has been able to predict the occurrence of earthquakes or the aftershock events [3]. Accordingly, it is essential to keep the bridges in service after ground motion excitations for emergency situations. Structural control systems have been used in various structures [4,5], as they are useful for reducing the vibrational response of structures with low lateral strength [6–8]. Several researchers have investigated the seismic and dynamic behavior of cable-stayed bridges [9–13]. The sources of geometric nonlinearity in cable-stayed bridges have been identified as the cable-sag effect, the large displacement effect, and the beam-column effect [14]. However, the cable-sag effect is considered to be the dominant source of nonlinearity in cable-stayed bridges. The dynamic behavior of the cable-stayed bridges is highly affected by the identified geometric nonlinearities [15].

The seismic behavior of cable-stayed bridges is significantly dominated by the connection of the deck with the tower and the piers [16]. During seismic events, the rigid connection limits the horizontal displacement of the deck, which causes the transmission of seismic forces from the superstructure to the substructure and increases the base shear of the tower [11,17,18]. In contrast, a floating or movable configuration increases the deck displacement and flexibility under service loadings [19,20]. Furthermore, the shape and height of the tower significantly influence the dynamic behavior of cable-stayed bridges [21–23].

A few researchers have studied the seismic control of cable-stayed bridges [12,24–26]. Ali and Abdel-Ghaffar [2] developed a constitutive model for cable-stayed bridges with a passive control system to achieve the optimal mechanical properties of the bearings. Wesolowsky and Wilson [27] focused on the base shear reduction of isolated cable-stayed bridges for near-field ground motions. In addition, the characteristics of near-field ground motions have been taken into account while designing the base isolators. Seismic isolation systems are an efficient control system for protecting new or for the retrofitting of existing bridges in seismic zones, as they are more economical compared to the conventional retrofitting of bridges [28–31]. Martínez-Rodrigo and Filiatrault [32] investigated the passive control and seismic isolation systems retrofitting alternatives for a two-dimensional model of a cable-stayed bridge in the longitudinal direction. The results confirmed that the implementation of friction pendulum bearings and fluid viscous dampers at the deck-tower connection are the best retrofitting solution among the passive control alternatives. Javanmardi et al. [33] thoroughly studied the characteristics of a base isolated cable-stayed bridge subjected to moderate and strong earthquakes and showed that the isolation system enhanced the overall seismic performance of the bridge. However, the isolation system induced a torsional plane at deck level, which resulted in an increment in the tower axial force on the substructure. Casciati et al. [34] assessed the seismic reliability of a retrofitted cable-stayed bridge equipped with hysteric devices. Fragility curves were developed to evaluate the performance threshold of structural members based on time history analysis. The control strategy overcame the effect of the uncertainties involved in the retrofitting strategies. Nevertheless, there is a lack of research on the seismic behavior of isolated cable-stayed bridges with distinctive features, such as a difference in the elevation of the abutments and irregularity of the geometry [24,25,35][36].

This paper studies the seismic response enhancement of an existing metallic cable-stayed bridge using the seismic isolation system. One of the four anchorage plates of the bridge supports failed during the 1988 Saguenay earthquake with a magnitude of M_L_ = 6.0 and peak ground acceleration of 0.15g. An earlier investigation proved that the failure was due to the high concentration of stresses under dead load and the stresses induced by seismic force [37,38]. The bridge was repaired immediately. Further, this bridge is asymmetric in the longitudinal direction with a difference in elevation between the end abutments of 7.3m. Hence, to prevent this sort of damage to the bridge in future, a seismic isolation system is introduced to protect the superstructure from the seismic load transmits from the substructure and to prevent the brittle failure of the supports. Five different base isolator combinations for the retrofitting schemes are considered in the numerical model to identify the best solution for the seismic performance enhancement of the bridge in both the longitudinal and transverse directions. The isolation systems are designed according to AASHTO [39,40]. The bridge is modeled precisely in three-dimensions including all the sources of nonlinearity and validated by the experimental results. The evaluation of each retrofitting configuration is performed using nonlinear time history analysis in the longitudinal and transverse directions.

## Methodology

### Description of Shipshaw cable-stayed bridge

The Shipshaw bridge is an asymmetric cable-stayed bridge with two planes of cables arranged in a fan shape spanning the Saguenay river, which is located in Canada. The bridge is made of a double leg steel tower and a composite deck supported by two box girders. The overall length of the bridge is 183.2m with four identical spans and a 4% downward slope from the East to the West abutment in the longitudinal direction. The bridge site is classified as rock. The tower base is hinged, which allows rotation in the longitudinal direction. The bridge abutments are roller supported to allow for longitudinal displacement as well as to withhold the uplifting of the bridge deck exhibited by the cable forces. The connection between the tower and the deck is a rigid connection.

**Fig 1:**
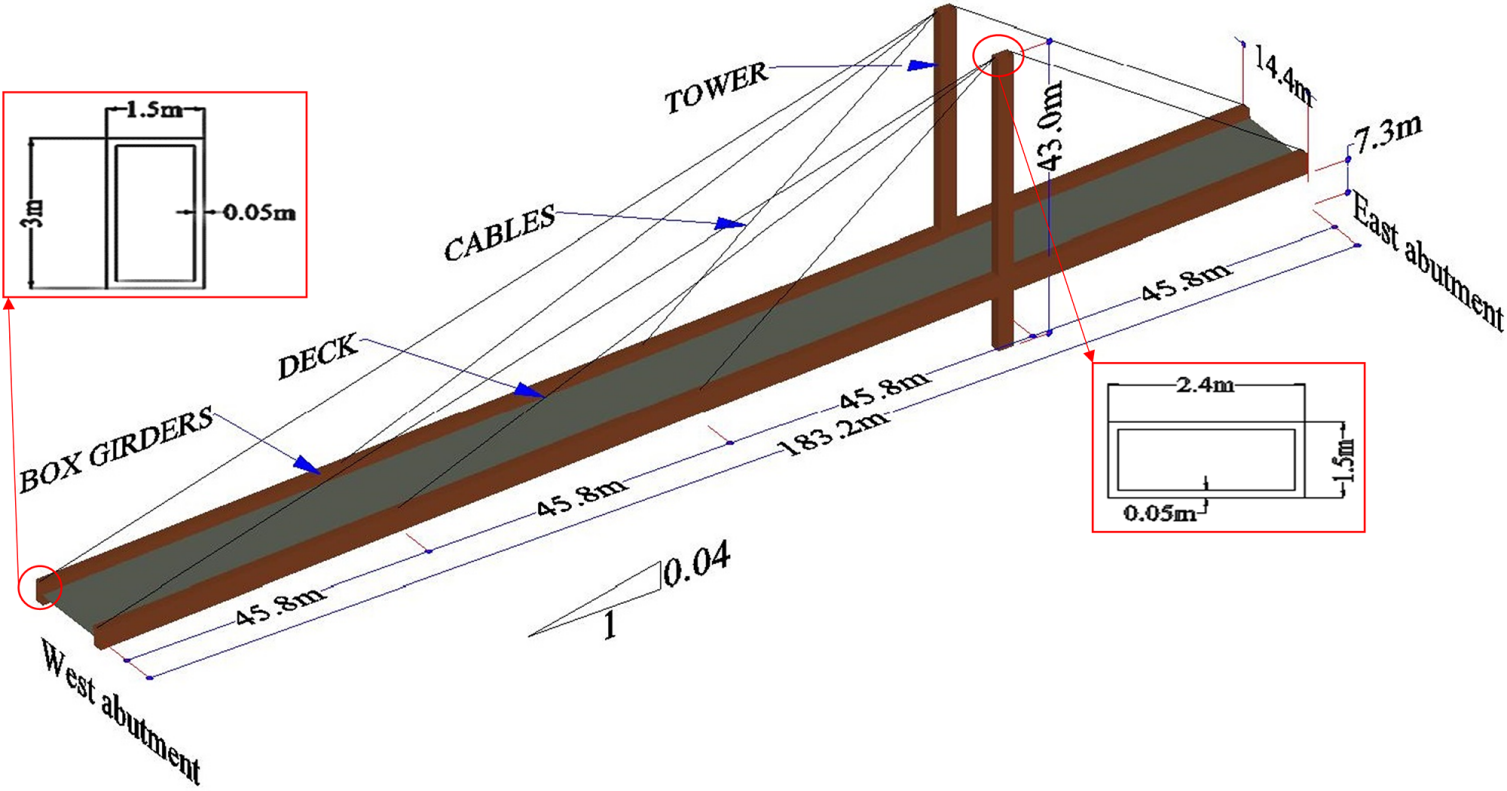
Shipshaw cable-stayed bridge detailing.

The deck has an 11 m wide concrete slab that is 165 mm thick with two non-structural precast parapets on the sides. In addition, five longitudinal stringers support the deck at equal intervals of 2.4 m. Floor beams transfer the stringer loads to the box girders and are spaced equally at 7 m intervals in the transverse direction. The dimensions of the box girder are 1.5 x 3 m with a web and flange thickness of 50 mm. The tower is 43 m tall and consists of two 2.4 x 1.5 m rectangular box girders with a flange and web thickness of 50 mm. The box girders and tower sections are stiffened with several stiffeners at certain distances to prevent both the global and local buckling due to axial forces. Each tower is connected by four cables to the top flange of the box girders at equal intervals. Each cable comprises nine strands of 65.1 mm^2^ cross-sectional area. Fig 1 shows the geometric detailing of the bridge.

### Numerical bridge modelling and validation

**Fig 2:**
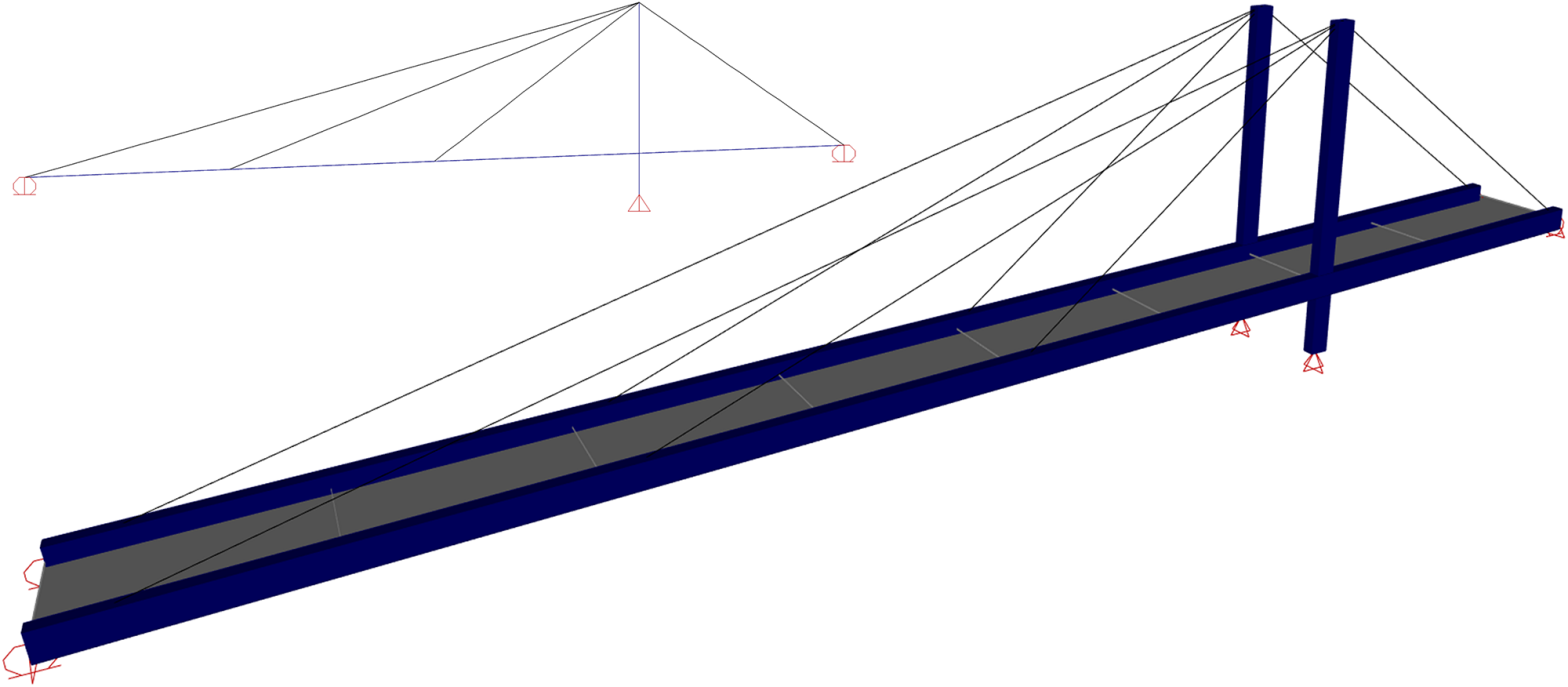
Finite element model of cable-stayed bridge.

The bridge is modeled and analyzed using SAP2000 finite element software [41]. The modal and nonlinear time history analyses are conducted on a three-dimensional full-scale model of the bridge; as illustrated in Fig 2. The 0.165 m thick concrete slab is modeled as a shell element. The tower and box girders are modeled as 3D beam elements. The tensile strength of the steel members is 414 MPa with a modulus of elasticity of 200 GPa. The compressive strength of the concrete deck is 27.5 MPa and it has an elastic modulus of 24.8 GPa. The cables are modeled as a cable element with an area of 585.9 mm^2^. A Young`s modulus of 175 GPA with a yield and ultimate strength of 1500 MPa and 1725 MPa, respectively, are assigned to the cables [42]. The pre-tensioning force of each cable is assigned to prevent the deflection of the deck under dead load. The cable element is able to model the catenary behavior of the cable under its self-weight. The tower base is hinged, which permits the tower to rotate along its longitudinal and transverse axes. At the abutments, the bridge can move freely along its longitudinal axis and it is restrained in both the transverse and vertical directions.

The failure criteria for the box girder and tower are defined as a series of plastic hinges with different properties for the frame elements. A 3D P-M2-M3 interaction surface is considered for the tower section. The P-M2-M3 hinge property represents the combined axial load and biaxial-bending moment behavior of the tower. A 2D M2-M3 interaction surface is considered for the box girder. The M2-M3 hinge property represents the biaxial bending moment behavior of the box girder. The moment-rotation interaction curves for each member are calculated using section designer in SAP2000. For all the hinges, the relevant hinge length is set to be 90% of the section depth. Since the cables are always modeled as tension members only, the failure criterion of this element is the elongation of the member up to rupture point, which is set to be 3.5% of the total length. Once the hinge reaches its maximum load carrying capacity it drops to zero. The static nonlinear analysis under the self-weight of the bridge is performed considering the material and geometrical nonlinearity to simulate the nonlinearity behavior of the cable-stayed bridge, which is followed up by modal analysis.

**Fig 3:**
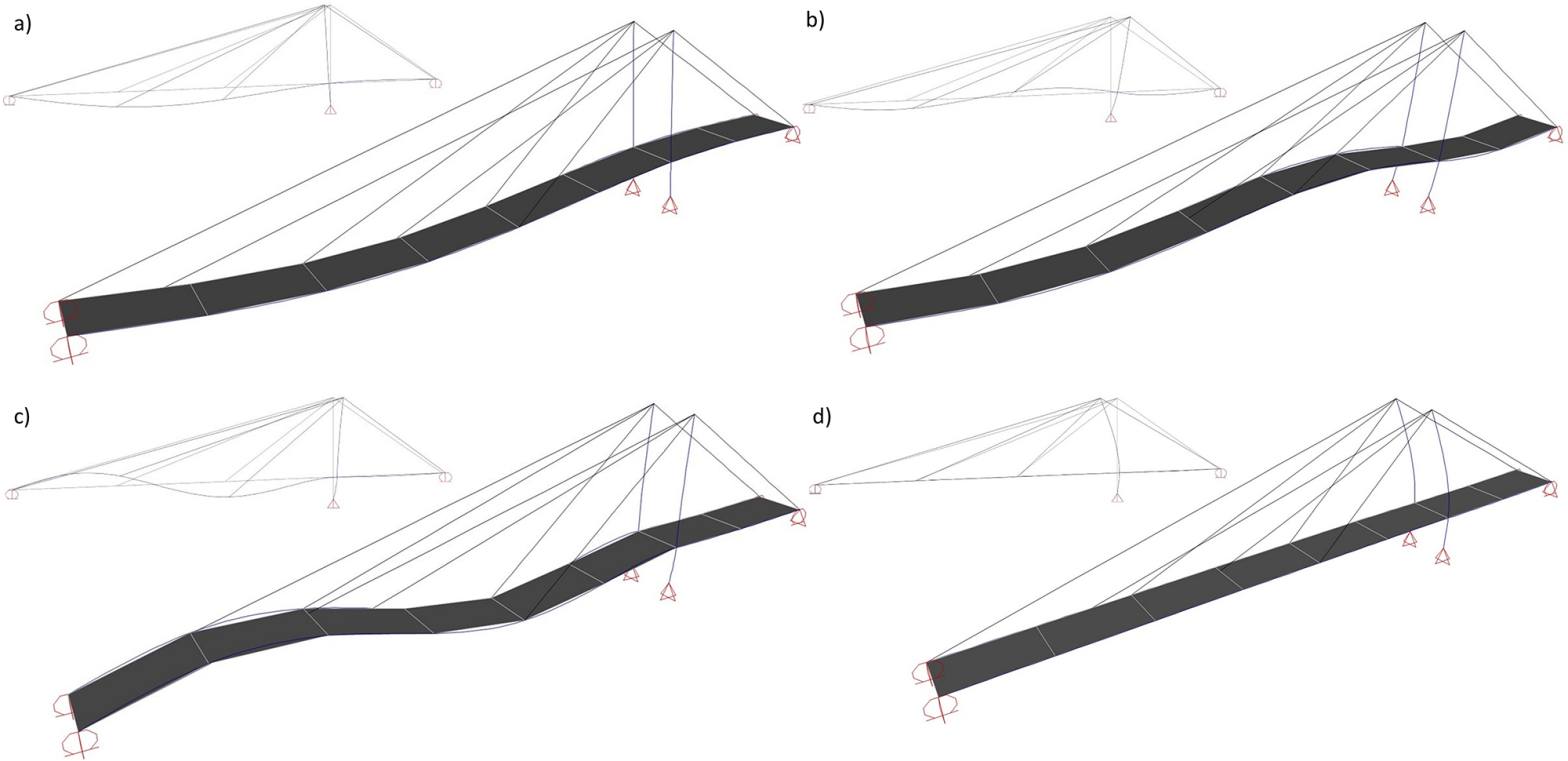
The cable-stayed bridge flexural mode shapes, a) 1^st^ flexural mode, b) 2^nd^ flexural mode, c) 3^rd^ flexural mode and d) 4^th^ flexural mode.

A full-scale field vibration test was conducted on the bridge using seven accelerometers. The accelerometers were placed on the top flanges of the box girders. A 44-ton truck with a constant speed of 80 km/h was used as the source of excitation. The rolling and break tests were performed to vibrate the bridge at different frequencies. The ULTRA [43] signal processing software was used for spectral analysis. The peak picking method was used to find the modal parameters from the Fourier spectrum. More details of the experiment test may be found in the research of Filiatrault et al. [38]. In the present study, a numerical analysis is carried out and verified by experiment. Fig 3 shows the four flexural mode shapes of the cable-stayed bridge from the numerical analysis. The sum of the modal mass of the first four flexural modes is 95.3% of the total mass of the bridge. The dominating flexural mode is the second mode with a mass participation percentage of 64.27%. The four time periods of the bridge from the experiment [38] and numerical modal analysis are illustrated in Table 1. As the table illustrates, the time periods of the cable-stayed bridge from the experiment and numerical analysis have a reasonable correlation.

**Table 1:** Flexural time periods of Shipshaw cable-stay bridge.

Mode shape	Deck Characteristic	Time periods (s)		Percentage difference (%)
		Experimental	Numerical	
First Flexural	Vertical/asymmetric	1.85	2.09	12.9
Second Flexural	Vertical/asymmetric	0.85	0.86	1.2
Third Flexural	Vertical/asymmetric	0.57	0.57	0.0
Fourth Flexural	Vertical/asymmetric	0.38	0.42	10.5

**Fig 4:**
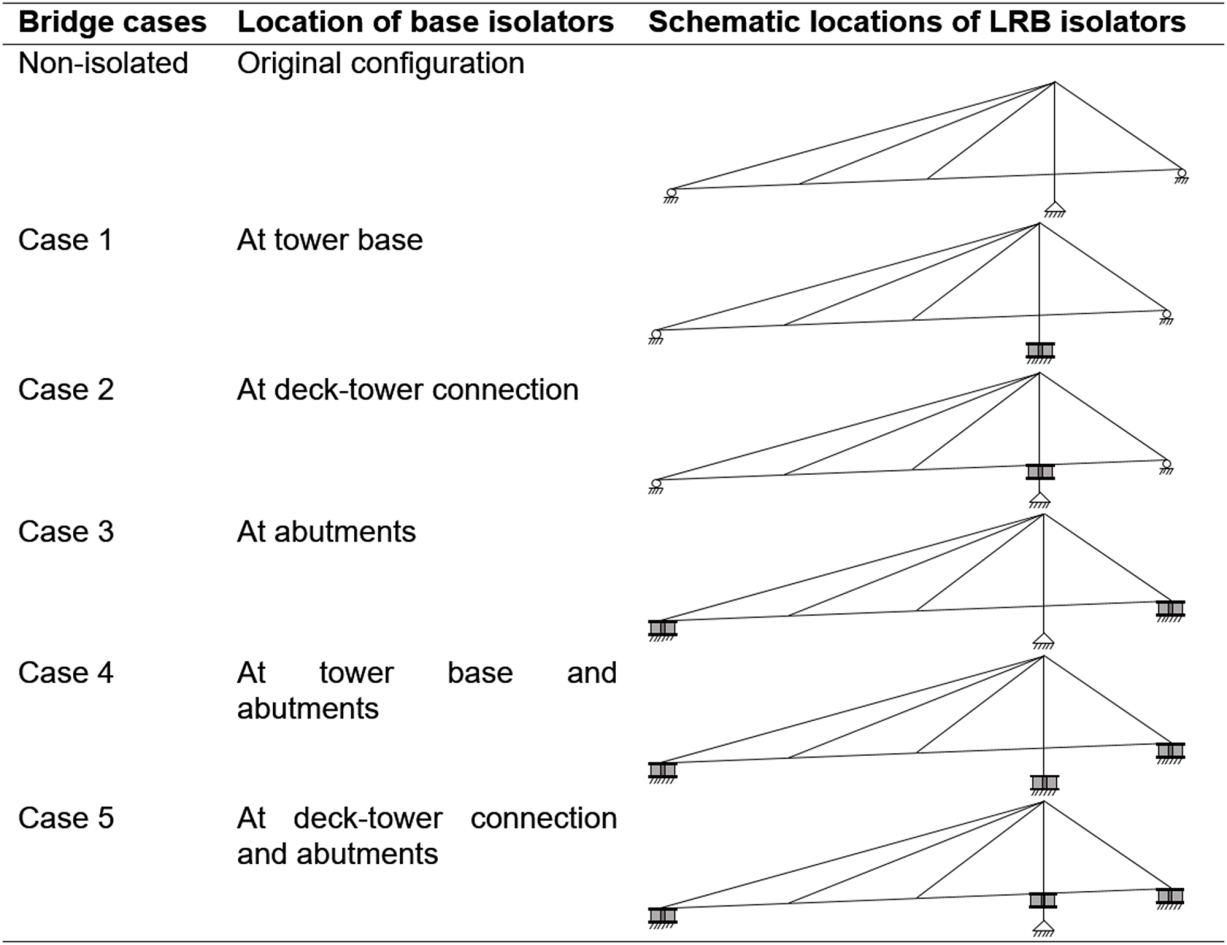
Different bridge cases with schematic locations of the isolation systems.

Generally, the isolation system separates the superstructure from the substructure at the deck level. As mentioned earlier the deck-tower connection greatly affects the dynamic behavior of the cable-stayed bridge. At the deck-tower connection, it is possible to implement isolators by permitting the deck to sit on the base isolators. Nonetheless, the base isolator can be used below the tower base by separating the tower legs from the foundation. However, the possibility of implementing base isolators at the tower base has not been taken into consideration in practice. Accordingly, a total of five combinations of the base isolators are found to be possible to identify the appropriate retrofitting solution for the cable-stayed bridge. Fig 4 illustrates the non-isolated and isolated cases of the bridge with schematic locations of the isolation systems.

### Earthquake ground motions

**Fig 5:**
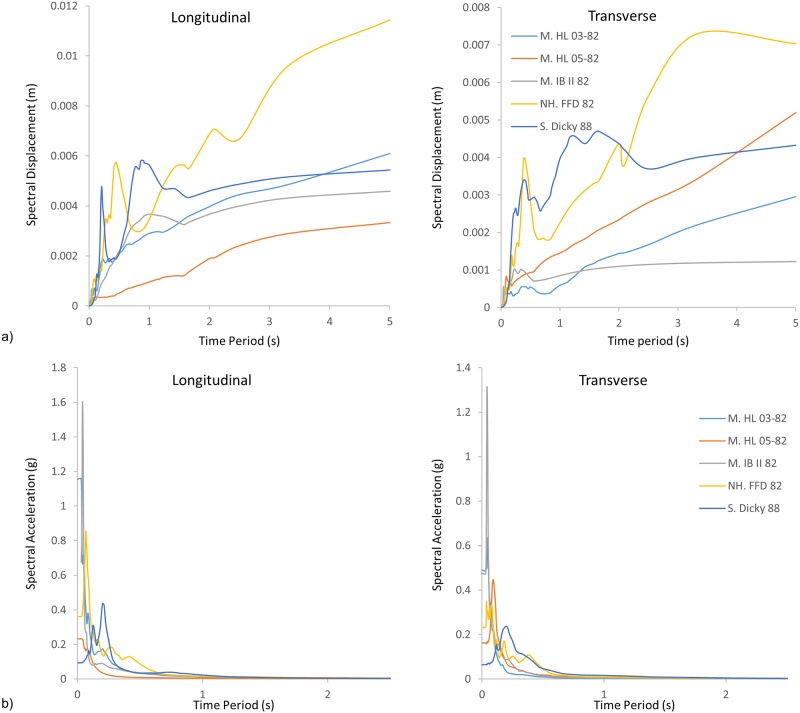
a) Spectral displacement and b) spectral acceleration of five earthquakes applied in longitudinal and transverse directions for the cable-stayed bridge with 5% damping.

After validation of the bridge model by the experimental results, the nonlinear time-history analyses of the modeled bridges are performed under different ground motions. Five ground motion records are selected to assess the seismic performance of the retrofitting strategies of the bridge. Each ground motion has two components in which the component with the greater PGA is applied in the longitudinal direction, and the component with a smaller PGA is applied in a transverse direction. One of the selected ground motion records corresponds to the actual ground motion that damaged the bridge, while the other four ground motion records are selected from the same seismic zone; as shown in Table 2. The computed response acceleration and displacement spectra for 5% damping are shown in Fig 5. This figure helps understand the energy-containing frequency of each ground motion employed in the analysis. The earthquakes are applied uniformly along all the supports, and the interaction of the soil-support is neglected.

**Table 2:** Ground motion characteristics.

Notation	Earthquake	Station	Country	Magnitude	Distance (Km)	Direction	PGA (g)	G_rms_ (g)	I_A_	HI (cm)	T_s_ (Sec)
M. HL 03–82	Miramichi-1982/03	Hickey Lakes-Site 3	Canada	5	6.5	Longitudinal	0.397	0.044	0.132	5.39	0.82
						Transverse	0.186	0.031	0.067	1.21	1.49
M. HL 05–82	Miramichi-1982/05	Hickey Lakes-Site 3	Canada	3.9	6.5	Longitudinal	0.111	0.011	0.017	1.33	0.69
						Transverse	0.110	0.007	0.006	2.82	0.96
M. IB II 82	Miramichi-1982/03	Indian Brook II	Canada	5	5.1	Longitudinal	0.342	0.042	0.124	4.21	0.28
						Transverse	0.290	0.035	0.084	1.68	0.33
NH. FFD 82	New Hampshire-1982	Franklin Falls Dam	USA	4.5	10.4	Longitudinal	0.313	0.016	0.076	7.37	2.63
						Transverse	0.126	0.010	0.031	4.54	7.68
S. Dicky 88	Saguenay-1988	Dicky	Canada	5.9	194.7	Longitudinal	0.092	0.005	0.070	6.83	21.51
						Transverse	0.063	0.003	0.036	5.63	41.62

PGA = Peak Ground Acceleration

G_rms_ = Root-Mean-Square Acceleration

I_A_ = Arias intensity

HI = Housner intensity

T_s_ = Significant duration

### Base isolator design

**Fig 6:**
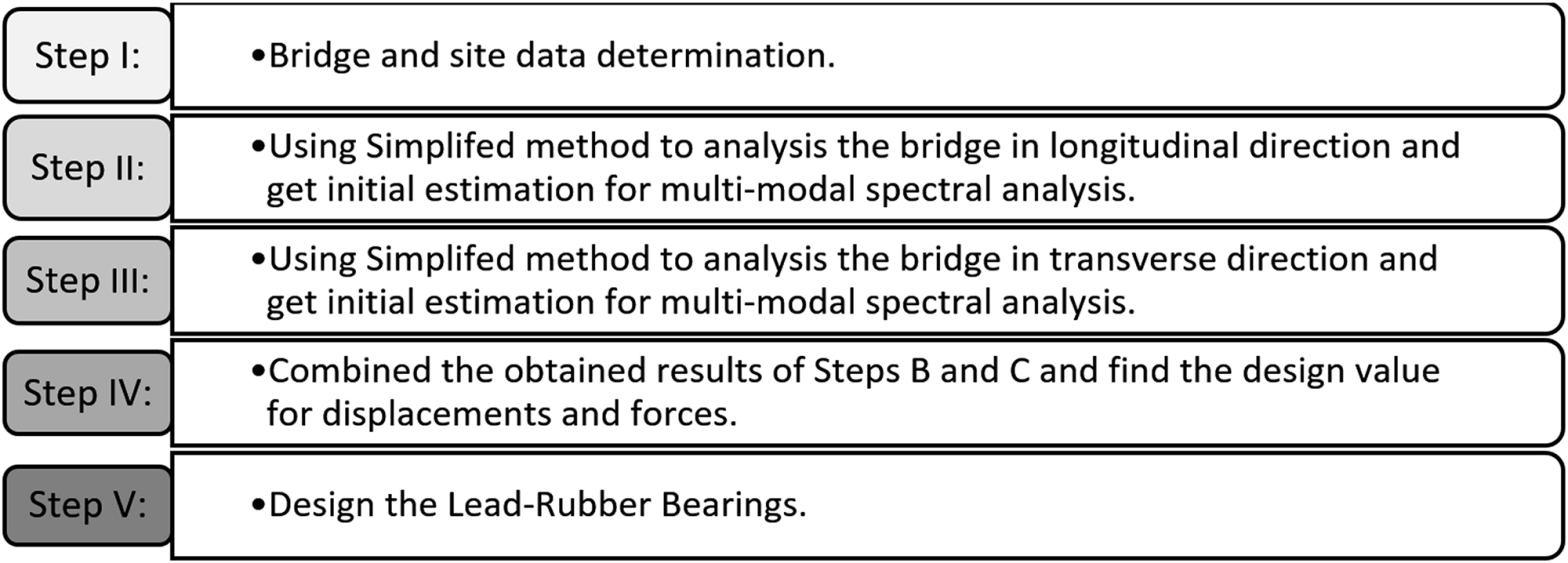
Design flowchart of the seismically isolated bridge.

**Fig 7:**
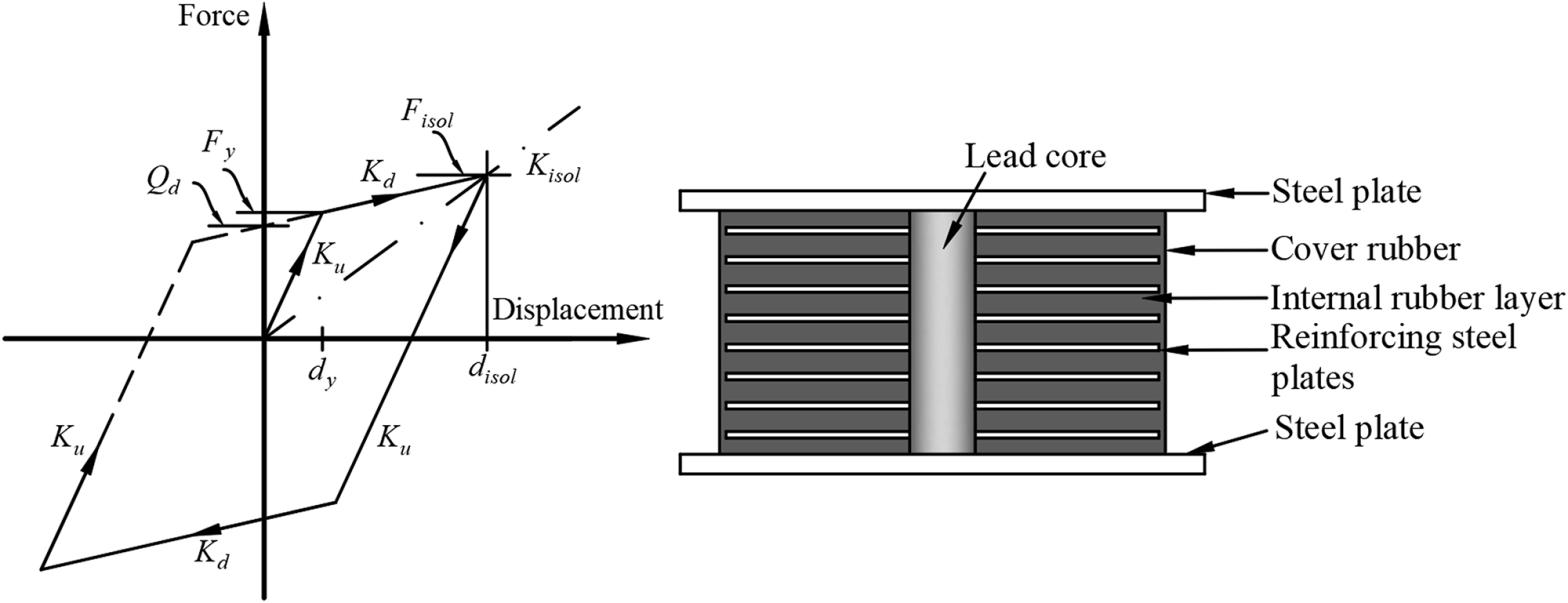
The detailing and hysteresis loop of the Lead Rubber Bearing (LRB). (*d*_*isol*_ = Isolator displacement, *d*_*y*_ = Isolator yield displacement, *F*_*isol*_ = Isolator shear force, *F*_*y*_ = Isolator yield force, *K*_*d*_ = Post-yield stiffness of isolator, *K*_*isol*_ = Effective stiffness of isolator, *K*_*u*_ = Loading and unloading stiffness (elastic stiffness), *Q*_*d*_ = Characteristic strength of isolator).

Robinson and Tucker [44] invented Lead-Rubber Bearing (LRB) in 1977, since when its application has gained popularity in civil structures located in seismic zones. In this study, the LRBs are designed according to the Guide Specifications for Seismic Isolation Design (GSID) AASHTO, 2010 [39] and the LRFD Bridge Design Specification (LRFD) AASHTO, 2012 [40]. The design methodology consists of five steps; as shown in Fig 6. Initially, the static analysis of the cable-stayed bridge under its self-weight is performed. The Bridge Site seismic specification is used in determining the i) seismic zone, ii) site class, and iii) acceleration coefficients and site factors. Then, the bridge is analyzed using the simplified method in both directions. The simplified method consists of several iterative processes to obtain convergence. The bilinear hysteretic behavior of the LRB isolator can be drawn from the design values; as illustrated in Fig 7. The initial bridge displacement, which is based on the SI system, can be assumed to be:

$$d=0.254S_{D1}$$ (1)

Where *S*_*D1*_ is the design spectral displacement. The characteristic strength, *Q*_*d*_, should be selected so that the isolator is stiff for non-seismic forces but yield under earthquake forces; hence, Eq 2 is found to be suitable for this purpose:

$$Q_{d}=0.05W$$ (2)

Where the weight of the superstructure on each isolator is W. The post-yield stiffness, *K*_*d*_, is defined as the minimum lateral restoring force at the design displacement, which can be obtained by:

$$K_{d}=0.05\frac{W}{d}$$ (3)

The effective period and the viscous damping ratio of the bridge are calculated according to Eqs 4 and 5, respectively:

$$T_{eff}=2\Pi \sqrt{\frac{W}{gK_{d}}}$$ (4)

$$\zeta =\frac{2Q_{d}(d_{isol}-d_{y})}{\Pi {\left(K_{eff}(d_{isol}+d_{sub})\right)}^{2}}$$ (5)

Where *d*_*isol*_, *d*_*y*_ and *d*_*sub*_ represent the isolator displacement, isolator yield displacement, and substructure displacement, respectively. Therefore, the total displacement of the bridge is:

$$d=\frac{0.254S_{D1}T_{eff}}{B_{L}}$$ (6)

Where *B*_*L*_ is the damping coefficient. The total displacement and the initial assumed displacement should have a close correlation. The iterative process in the spreadsheet is used to achieve this.

$$F_{isol}=K_{isol}\times d_{isol}$$ (7)

#### Biaxial model for Lead Rubber Bearings

The forces mobilization in the isolators with biaxial interaction can be calculated by [45]:

$$F_{2}=\alpha \frac{F^{Y}}{Y}U_{2}+(1-\alpha )F^{Y}Z_{2}$$ (8)

$$F_{3}=\alpha \frac{F^{Y}}{Y}U_{3}+(1-\alpha )F^{Y}Z_{3}$$ (9)

**Fig 8:**
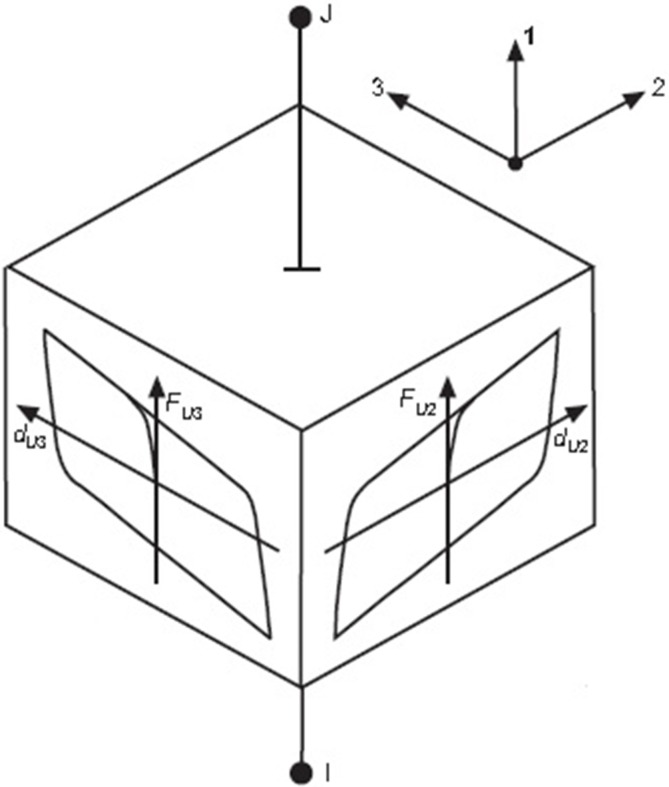
The schematic hysteretic behavior of LRB with biaxial shear deformation [46].

Where α, F^Y^ and Y are the post-yield stiffness to pre-yield stiffness ratio, the yield force, and the yield displacement, respectively. U_2_ and U_3_ are the displacements of the isolators in the local direction, as depicted in Fig 8 (2 and 3 directions); while Z_2_ and Z_3_ are the non-dimensional quantities that represent the direction and biaxial interaction of the hysteretic forces. Z_2_ and Z_3_ are calculated by the coupled differential equations [46]:

$$Y\left(\begin{array}{c} {\dot{Z}}_{2}\\ {\dot{Z}}_{3} \end{array}\right)=A\left(\begin{array}{c} {\dot{U}}_{2}\\ {\dot{U}}_{3} \end{array}\right)\left[\begin{array}{cc} Z_{2}^{2}\left[\gamma sign({\dot{U}}_{2}Z_{2})+\beta \right] & Z_{2}Z_{3}\left[\gamma sign({\dot{U}}_{3}Z_{3})+\beta \right]\\ Z_{2}Z_{3}\left[\gamma sign({\dot{U}}_{2}Z_{2})+\beta \right] & Z_{3}^{2}\left[\gamma sign({\dot{U}}_{3}Z_{3})+\beta \right] \end{array}\right]$$ (10)

Where A, β, and γ are dimensionless quantities. The LRB in SAP2000 software is modeled using the nonlinear link element to produce its orthotropic behavior when α and the yield force vary in 2 and 3 directions (the local directions).

## Results of modal analysis

### Natural time period of the bridge

The natural time period and the relevant mass participation ratio of the bridge for non-isolated and different isolated cases from modal analysis are illustrated in Table 3. The natural time period of the retrofitted bridge should not exceed 1.7 times the original natural time period as it increases the seismic displacement response of the bridge [20]. The isolation system has no significant effect on the natural time period of the bridge in cases 1 and 2. However, in cases 3, 4, and 5 the isolation system lengthens the natural time period by 16.75%, 18.66%, and 43.54%, respectively. Therefore, the flexibility of the cable-stayed bridge is increased in cases 3, 4, and 5. The mass participation ratio of the bridge in all isolated cases is incremented in the range of 82.68% to 92.99%. As a consequence, the first mode becomes the main contributing mode of the bridge after the implementation of isolators in all retrofitting cases; as shown in Table 3.

**Table 3:** Fundamental period of the bridge.

	Non-isolated	Isolated-case 1	Isolated-case 2	Isolated-case 3	Isolated-case 4	Isolated-case 5
First time period (s)	2.09	2.11	2.09	2.44	2.48	3.00
Mass participation ratio (%)	2.16	92.99	89.60	82.68	89.93	92.62

**Fig 9:**
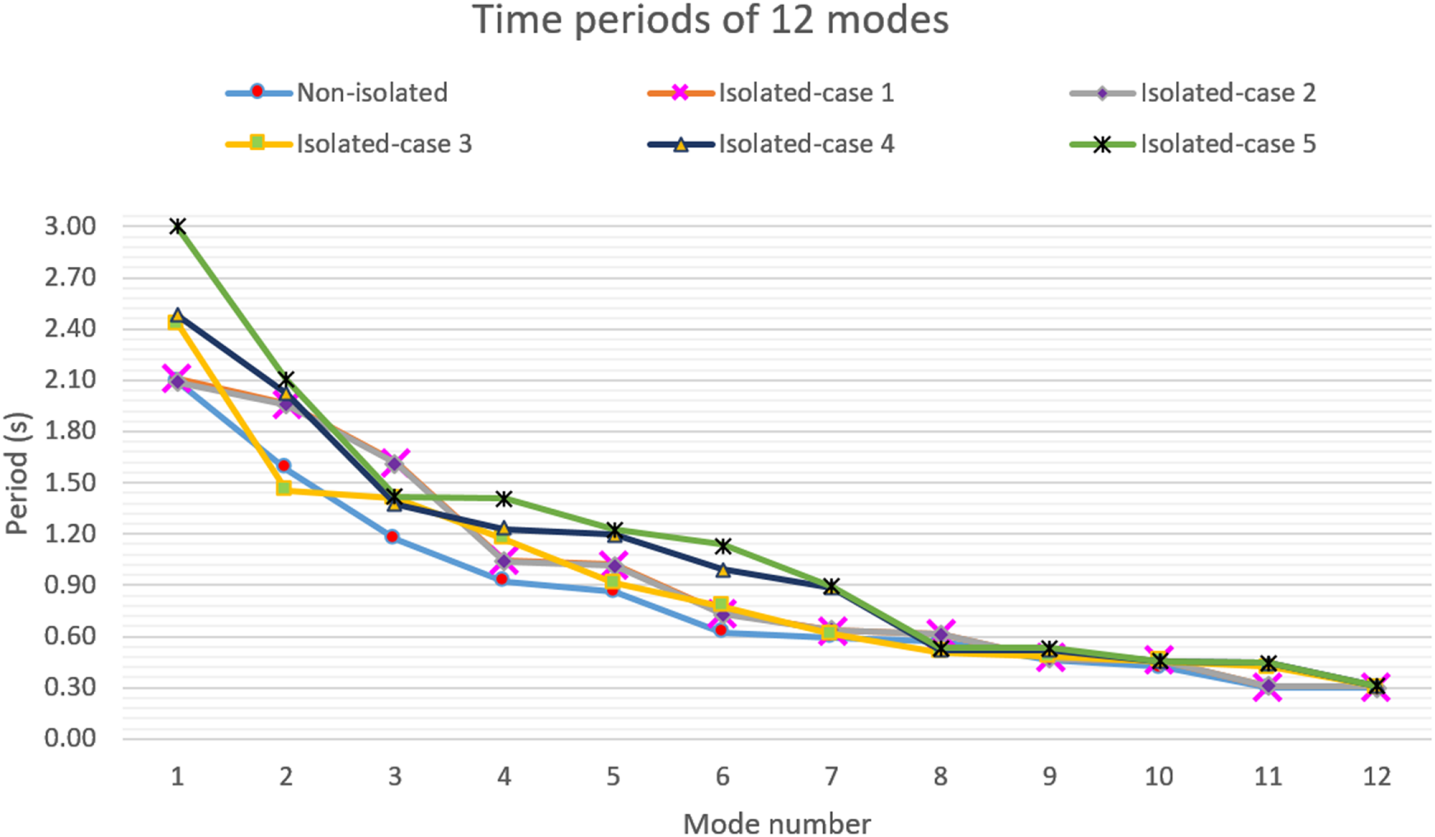
Implementation effect of base isolators on the natural time periods of the bridge.

The 12 modes versus the natural time periods of the bridge are illustrated in Fig 9. The implementation of base isolators led the bridge to have higher flexibility behavior than the original configuration. The dynamic behavior trend of the bridge is notably changed in the retrofitting cases, as the natural time periods are increased compared to the original bridge time period. For cases 1 and 2, the trend of the natural time periods is almost similar with consistent flexibility until the 7th mode of the bridge. Consequently, it can be seen that these two have similar seismic responses, in which the isolators are utilized along the tower base and deck-tower connection. Furthermore, the highest impact is seen for case 5 where the fundamental period is increased by 43.54%. These dynamic changes in the bridge are favorable, which leads to a reduction in the seismic forces transmitted from the substructure to the superstructure.

## Results of time-history analysis

### Bridge displacement

**Fig 10:**
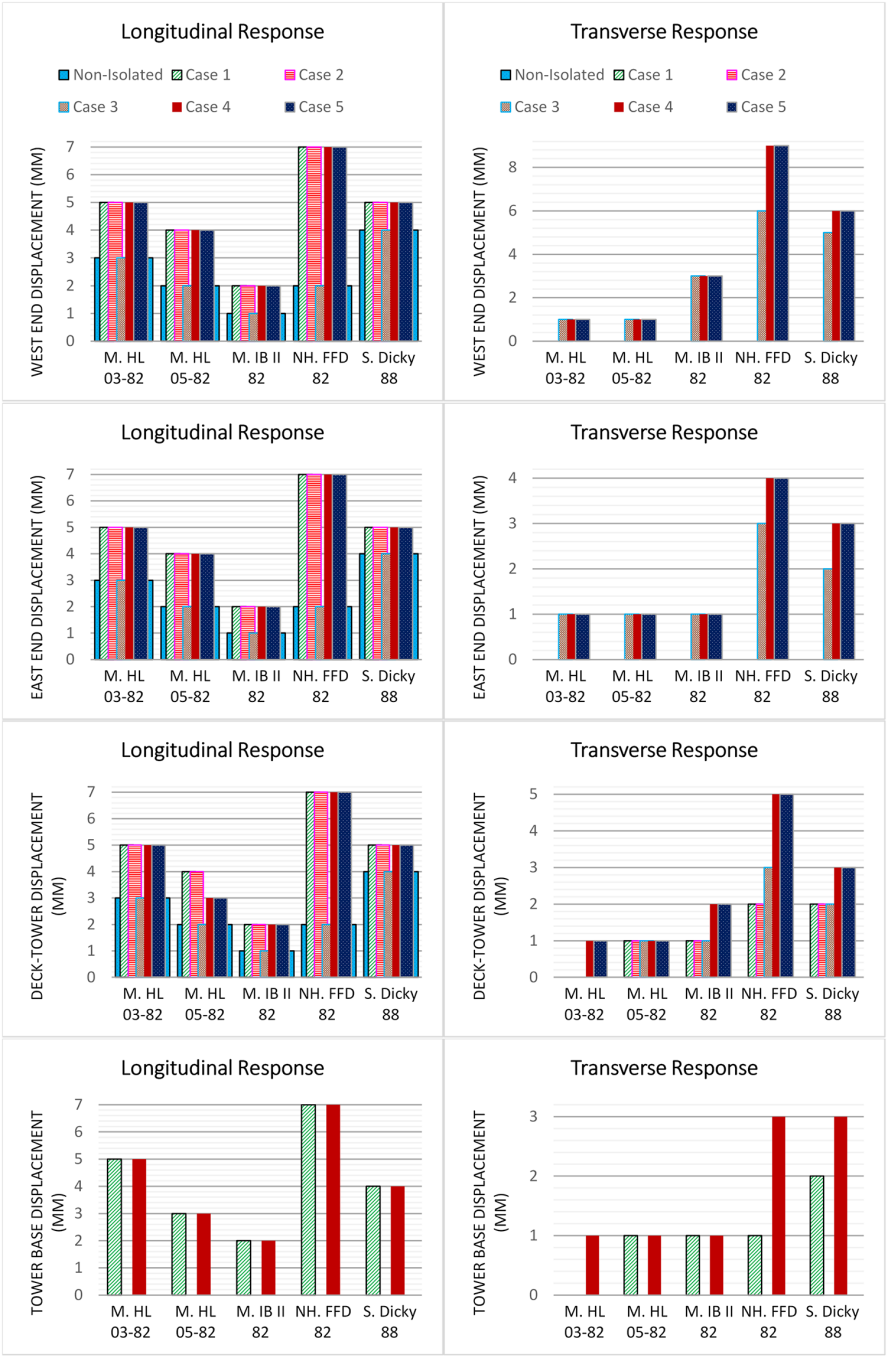
Maximum bridge displacement under earthquake excitations.

Fig 10 shows the bridge displacements at the tower base, deck-tower connection, and West and East ends for all the cases. When the isolation system is implemented at the base of the tower in cases 1 and 4, the displacements in the longitudinal and transverse directions are observed. The maximum tower displacements in these cases are 7 mm and 3 mm under the NH. FFD 82 earthquake in the longitudinal and transverse directions, respectively. The bridge displacement at the deck-tower connection is increased in the longitudinal direction in all seismic isolation cases except case 3. Under the NH. FFD 82 earthquake, the longitudinal displacement of this point is increased by 250%. For this point, the transverse displacement is recorded for cases 1, 2, 3, 4, and 5. The maximum transverse displacement of the deck-tower connection is 5 mm due to the NH. FFD 82 earthquake. In the longitudinal direction, the bridge ends in the initial configuration were free to move. After the implementation of the isolation systems, the bridge end displacements increases in all cases except for case 3. In case 3, the isolation system is installed at the bridge ends, while the deck-tower connection and tower base has the same configuration as the non-isolated. The displacement of the West and East ends of the bridge is increased up to 250% under NH. FFD 82 in cases 1, 2, 4, and 5. In the transverse direction for non-isolated case 1 and case 2, zero displacement is observed as the bridge is restrained in this direction. After the implementation of the isolators at the bridge ends, transverse displacements are detected in cases 3, 4, and 5. The maximum displacement increment for both the West and East ends of the bridge is 50% recorded under the NH. FFD 82 and S. Dicky 88 earthquakes, respectively. The utilization of the seismic isolation system at the tower base or deck-tower connection has increased the longitudinal displacement of the bridge. Hence, the rigid connection of the deck and tower and the tower support condition are the dominating factors in controlling the longitudinal seismic displacement of the bridge. From Fig 10 it can be seen that the bridge displacements are relatively very small in both directions; up to a few millimeters even after the implementation of the isolation system. The bridge displacements are limited to the designed displacements obtained by the simplified analysis of the bridge in the design of the seismic isolation.

### Tower response

The key structural member in cable-stayed bridges is the tower as it supports all the stay cables. The failure or instability of the tower may cause the total collapse of the cable-stayed bridge. Hence, it is essential to understand the seismic responses of the tower under earthquake excitations. The results for the base shear and base moment responses of the tower under different bidirectional earthquake excitations are presented for this purpose.

#### Base shear

**Fig 11:**
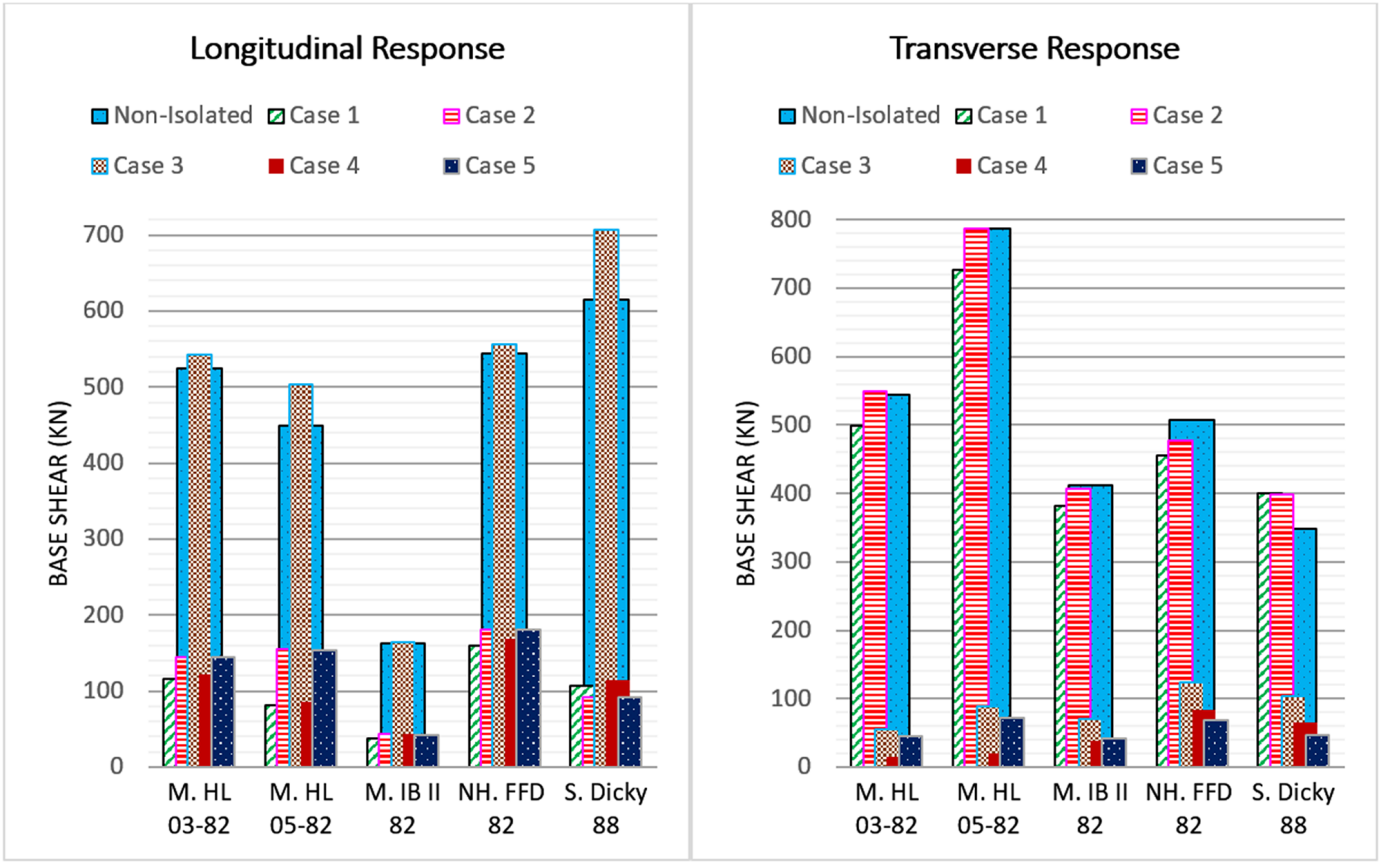
Maximum base shear response of towers subjected to earthquake excitations.

One of the main aims of seismic isolation is to minimize the base shear of the bridge under seismic excitations. The implementation of isolation systems may not always reduce the base shear in both directions; as demonstrated in Fig 11. As the results of the numerical study show, the base shear in cases 1 and 2 is reduced in the range of 65.53% to 85.04% in the longitudinal direction. However, in the transverse direction the base shear for cases 1 and 2 shows a significant increase. The maximum base shear increments observed in cases 1 and 2 are 15.10% and 14.42%, respectively, which occurred under the S. Dicky 88 earthquake. This is because, in the original configuration of the bridge, the tower is allowed to freely rotate in a transverse direction while the implementation of the isolators at the tower base and tower-deck connection restrains the transverse rotation of the tower through the stiffness of the isolators. In case 3, in the longitudinal direction, the base shear for all the earthquakes increases significantly. The base shear increases up to 14.9%. However, for this case, the base shear reduces up to 90% in the transverse direction. The non-isolated bridge supports at the abutments are free to move in the longitudinal direction but are restrained in the transverse direction; while, in case 3, the isolators restrain the longitudinal movement of the bridge but allow limited transverse movement. This led to an increment of the base shear in the longitudinal direction and a reduction of the base shear in the transverse direction. The base shear in both directions is reduced significantly for cases 4 and 5. In case 4, the maximum base shear reductions in the longitudinal and transverse directions are 81.47% and 97.44%, respectively, which occurred under the S. Dicky 88 and M. HL 05–82 earthquakes. In case 5, the base shear reduction is 85.15% in the longitudinal direction under the S. Dicky 88 earthquake, while the base shear is reduced by 91.7% in the transverse direction under the M. HL 03–82 earthquake. The results prove that the implementation of base isolators at one or two supports is insufficient to reduce the base shear in both directions. A remarkable base shear reduction is observed in the cases in which the isolation systems are implemented at three locations of the bridge simultaneously.

#### Base moment

**Fig 12:**
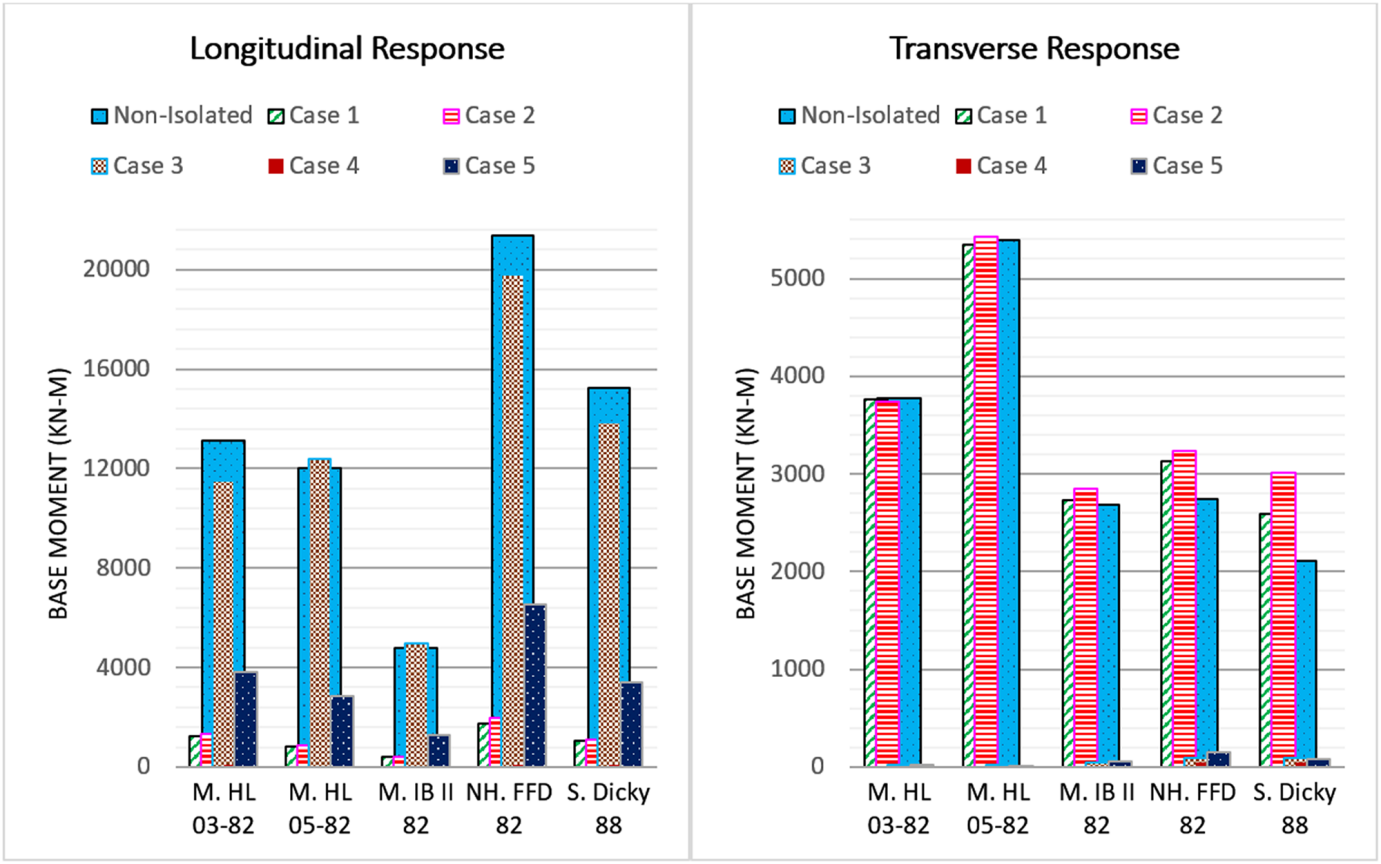
Maximum base moment response of tower subjected to earthquake excitations.

The maximum bending moments of the tower in both directions are presented in Fig 12. In the longitudinal direction, the base moment for all the cases significantly decreases except in case 3. The maximum base moment reduction is 99.54% in case 4 under the M. HL 05–82 earthquake. In contrast, the base moment in case 3 increases by 3% and 3.61% under M. HL 05–82 and M. IB II 82 ground motions, respectively. This is due to the change in the configuration of the bridge abutments with the base isolators. The roller supports of the original configuration have zero longitudinal stiffness, while, the isolated cases have stiffness.

In the transverse direction, the isolation system in cases 1 and 2 cause a notable increment in the base moment of the tower. The base moment increments are in the range of 2% to 42.6% since the original tower base is allowed to rotate freely in both directions but the implementation of the base isolators restricts the rotation due to their stiffness. The maximum base moment increment occurs in case 2 where it reaches 3011.7 kN-m under the S. Dicky 88 earthquake. Nonetheless, in cases 3, 4, and 5 the base moment reduces remarkably. In case 3, the base moment reduces by up to 99.6%, as the original configuration of the bridge is restricted from moving in a transverse direction at the abutments and because the base isolators omit this restriction with movement up to the design displacement. In case 4 the base moment is reduced to 9.7, 13.5, 12.2, 50.4 and 42 kN-m under M. HL 03–82, M. HL 05–82, M. IB II 82, NH. FFD 82 and S. Dicky 88 ground motions, respectively. Similarly, the percentage reduction in case 5 is 99.55%, 99.71%, 98.06%, 94.5% and 96.35% when the bridge is subjected to M. HL 03–82, M. HL 05–82, M. IB II 82, NH. FFD 82 and S. Dicky 88 earthquakes, respectively.

For cases 4 and 5, the base moment enhancement is more significant due to the combination of the isolation system along the bridge supports and the deck-tower connection. However, the case 4 performance is still more remarkable than case 5. The base moment reduction prevents the overturning moment of the tower during bidirectional earthquake excitations.

### Cable response

**Fig 13:**
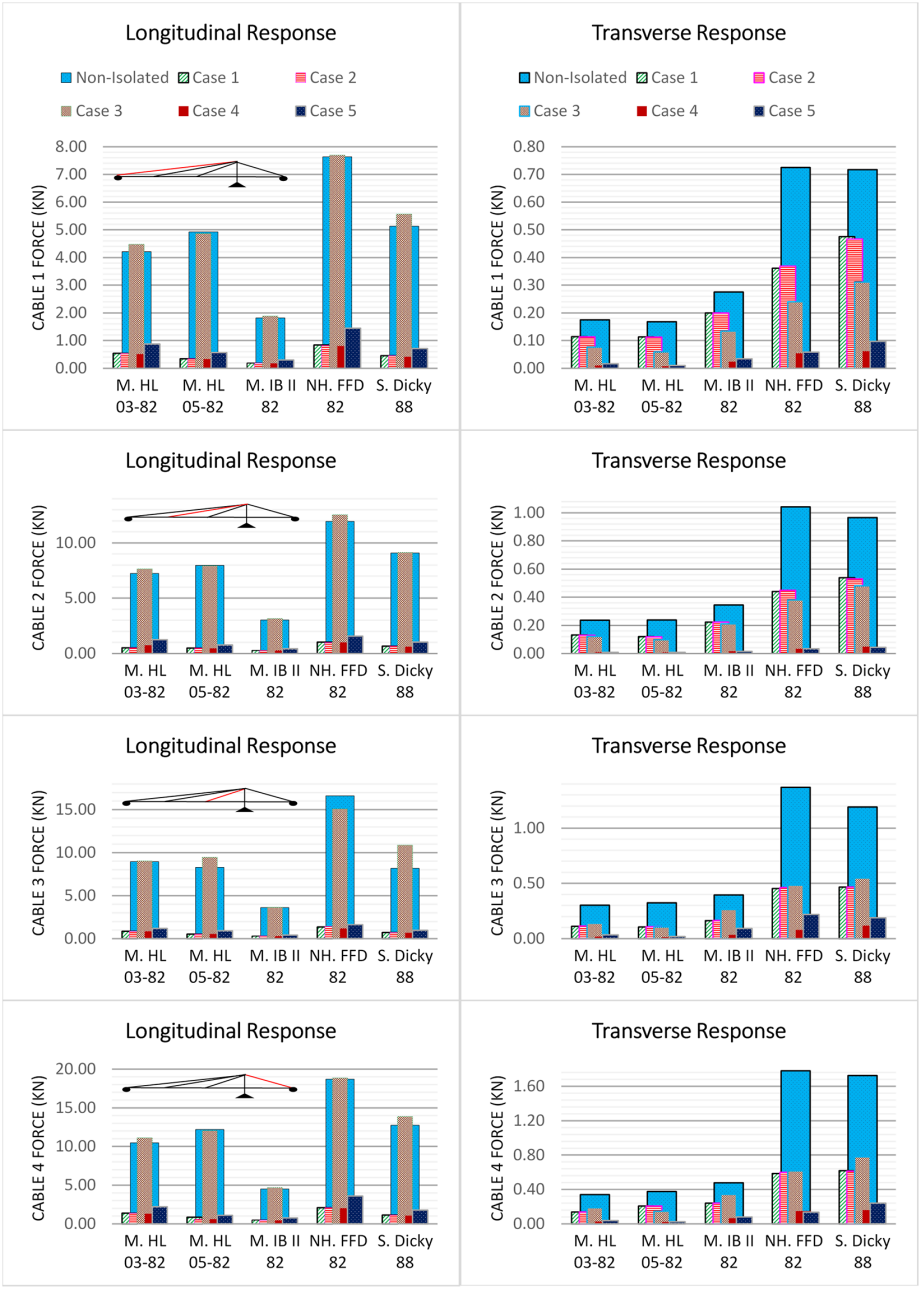
Maximum cable tension forces of the bridge under earthquake excitations.

Here, since in the transverse direction the bridge is symmetric, the cables on one side of the bridge are chosen for the investigation. The numbering of the cables from the West to the East abutments is 1, 2, 3, and 4, respectively. The cable force should remain within the nominal range and never approach zero. The cable force changes are with respect to their existing nominal pretension force; as illustrated in Fig 13. It can be seen from the figure that the cable forces vary during vibration of the bridge; these variations are within the range of 0.2T to 0.7T specified by Dyke et al. [47]. The implementation of base isolators causes a notable reduction in the variations in the force in all the cables for all cases except case 3. The cable force changes are enhanced up to 96.74% and 95.71% in the longitudinal and transverse directions, respectively. In the longitudinal direction for case 3, the cable forces increase up to 8.5%, 5.6%, 33.1% and 8.9% for cables 1, 2, 3, and 4, respectively. However, in the transverse direction, the cable forces in case 3 are considerably enhanced. Furthermore, the tension forces in cables 3 and 4 are larger compared to cables 1 and 2 under earthquake excitations, because they are connected to the box girder at closer distances to the tower.

### Hysteresis response of bridge

**Fig 14:**
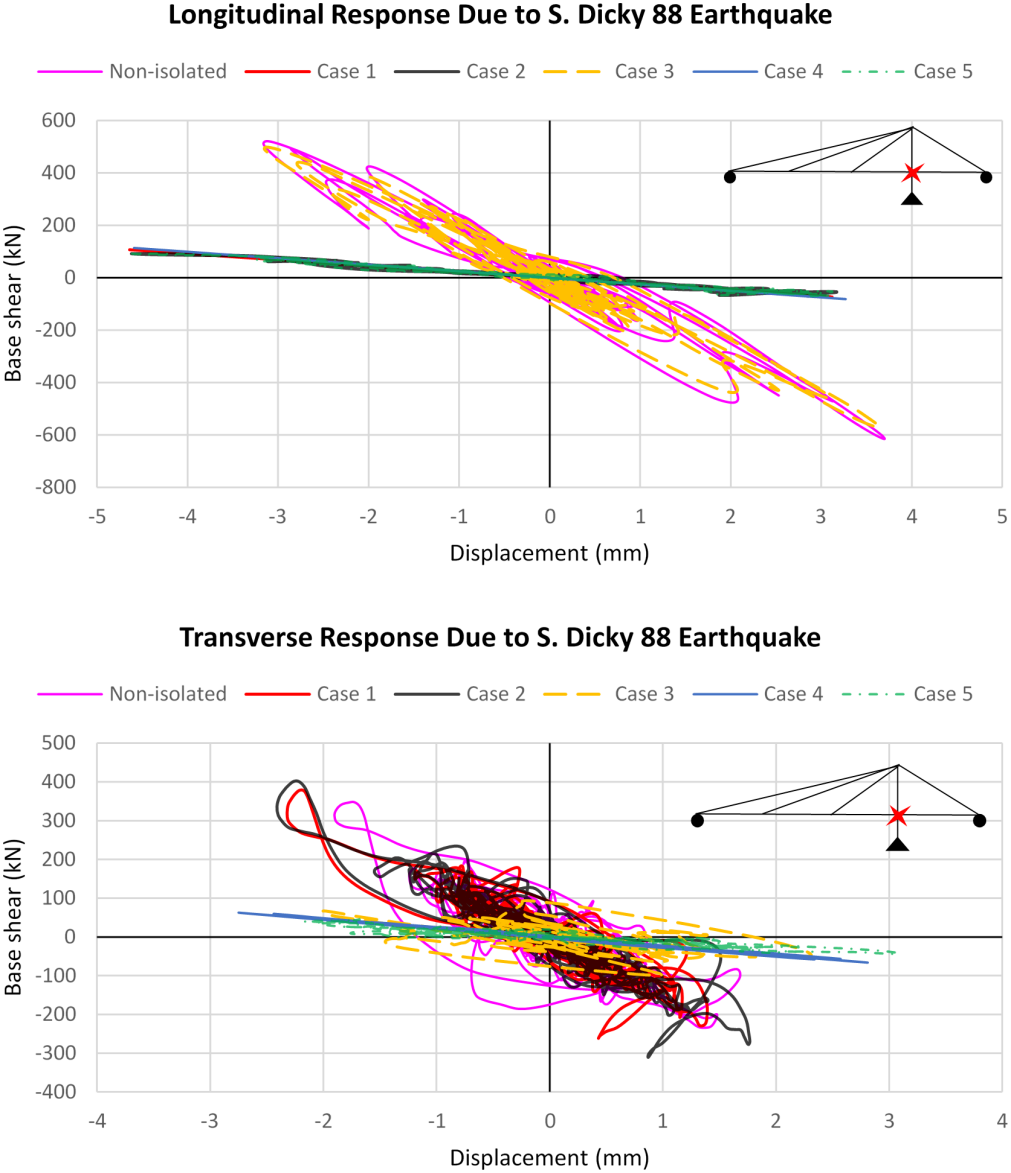
Force-displacement hysteresis curves of the bridge subjected to S. Dicky 88 earthquake.

In this section, the hysteresis behavior of the bridge for different cases is investigated. The S. Dicky 88 earthquake is the actual event that caused the failures in the bridge; therefore, to avoid too many hysteresis graphs, only the hysteric curves of the bridge under the S. Dicky 88 earthquake are presented in both directions. Fig 14 represents the hysteresis curves of the base shear of the tower versus the displacement of the bridge at the deck-tower connection. As the figure shows, the implementation of the isolation system causes a significant enhancement in the bridge response in the longitudinal direction. However, the improvement in the response of the bridge is not the same in all cases. In the transverse direction, the bridge response increases for cases 1 and 2, which have almost the same hysteric curves, while in other cases the bridge response improves through implementation of the isolation system. The original configuration of the bridge experiences a large number of yielding cycles, while the isolated bridge in cases 4 and 5 shows no inelastic cycles in either direction.

### Overall seismic response

The overall seismic responses of the cable-stayed bridge are summarized and presented in Table 4. However, the displacements of the bridge ends are not included since the displacement values are very small and readable in Fig 10. As the table indicates, cases 4 and 5 have similar seismic responses. These two cases are the most favorable retrofitting cases, in which the overall seismic performance of the bridge is enhanced substantially in both directions under all the earthquake excitations. Subsequently, in cases 1 and 2, the longitudinal performance of the bridge is improved while in the transverse direction no significant improvement is observed. Finally, the seismic performance of the bridge is only improved in the transverse direction for case 3.

**Table 4:** Summary of seismic responses of the bridge for different retrofitting cases.

Earthquake	Bridge Case	Base shear (kN)		Base moment (kN- m)		Cable 1 force (kN)		Cable 2 force (kN)		Cable 3 force (kN)		Cable 4 force (kN)	
		X	Y	X	Y	X	Y	X	Y	X	Y	X	Y
M. HL 03–82	Non-isolated	525.1	544.7	13147.1	3772.5	4.22	0.18	7.25	0.24	8.96	0.30	10.46	0.34
	Case 1	115.3	499.4	1239.7	3766.1	0.54	0.11	0.51	0.13	0.86	0.11	1.36	0.14
	Case 2	144.6	548.6	1325.3	3736.8	0.54	0.11	0.50	0.13	0.86	0.11	1.37	0.14
	Case 3	542.3	54.5	11534.3	19.1	4.48	0.08	7.65	0.12	9.04	0.14	11.11	0.19
	Case 4	121.2	14.8	77.5	9.7	0.52	0.01	0.76	0.01	0.86	0.02	1.32	0.03
	Case 5	143.9	45.2	3797.1	16.9	0.88	0.02	1.25	0.01	1.19	0.03	2.20	0.04
M. HL 05–82	Non-isolated	449.2	786.55	12033.1	5388.8	4.93	0.17	7.98	0.24	8.30	0.32	12.19	0.38
	Case 1	81.3	727.0	840.2	5351.2	0.34	0.11	0.50	0.12	0.52	0.11	0.87	0.21
	Case 2	154.8	787.3	861.7	5427.9	0.34	0.11	0.50	0.12	0.52	0.11	0.57	0.21
	Case 3	503.5	88.2	12391.2	23.1	4.86	0.06	7.94	0.10	9.48	0.11	12.04	0.15
	Case 4	86.4	20.1	54.9	13.5	0.33	0.01	0.48	0.01	0.56	0.01	0.61	0.02
	Case 5	154.3	71.1	2843.5	15.5	0.56	0.01	0.78	0.01	0.91	0.02	1.11	0.02
M. IB II 82	Non-isolated	162.9	412.5	4786.6	2688.1	1.81	0.28	3.03	0.34	3.63	0.40	4.51	0.48
	Case 1	37.0	382.0	418.5	2735.1	0.19	0.20	0.28	0.22	0.30	0.16	0.48	0.24
	Case 2	43.0	407.0	412.2	2848.7	0.19	0.20	0.28	0.22	0.30	0.17	0.48	0.24
	Case 3	164.3	69.9	4959.5	40.5	1.89	0.14	3.16	0.21	3.65	0.27	4.69	0.34
	Case 4	44.0	37.8	27.9	12.2	0.18	0.02	0.27	0.02	0.30	0.03	0.46	0.07
	Case 5	42.7	42.3	1299.3	52.2	0.30	0.03	0.44	0.02	0.42	0.09	0.76	0.08
NH. FFD 82	Non-isolated	544.7	508.1	21361.1	2744.1	7.63	0.73	11.93	1.04	16.62	1.37	18.72	1.78
	Case 1	160.3	454.9	1761.9	3124.5	0.84	0.36	1.02	0.44	1.34	0.45	2.10	0.59
	Case 2	181.5	476.9	1996.9	3231.5	0.82	0.37	1.00	0.45	1.40	0.46	2.07	0.60
	Case 3	555.8	123.0	19786.9	87.0	7.69	0.24	12.56	0.38	15.08	0.48	18.85	0.62
	Case 4	169.4	83.8	107.9	50.4	0.81	0.05	1.00	0.03	1.17	0.08	2.03	0.15
	Case 5	181.4	68.3	6543.3	151.0	1.45	0.06	1.56	0.03	1.63	0.22	3.59	0.14
S. Dicky 88	Non-isolated	615.0	347.9	15231.9	2112.4	5.13	0.72	9.09	0.97	8.18	1.19	12.47	1.73
	Case 1	107.0	400.5	1075.1	2591.3	0.45	0.48	0.66	0.54	0.71	0.47	1.14	0.62
	Case 2	92.0	398.1	1108.2	3011.7	0.46	0.47	0.66	0.53	0.72	0.47	1.14	0.61
	Case 3	706.6	103.1	13867.9	92.9	5.57	0.31	9.13	0.48	10.88	0.55	13.88	0.78
	Case 4	113.9	66.0	72.6	42.0	0.42	0.06	0.61	0.05	0.68	0.12	1.06	0.16
	Case 5	91.3	46.0	3418.1	77.1	0.71	0.10	1.02	0.04	0.98	0.19	1.77	0.24

## Summary and conclusions

This paper investigated the seismic performance of an existing steel cable-stayed bridge retrofitted with LRBs under bi-directional ground motions. The main objective was to find the most adequate seismic retrofit configuration for the cable-stayed bridge to enhance its longitudinal and transverse seismic performance. In line with this purpose, a three-dimensional finite element model of the bridge was created and analyzed through the nonlinear time-history analysis to evaluate each retrofitting configuration. The difference in the elevation of the abutments and sources of nonlinearity of the cable-stayed bridge were taken into account during the analysis. From the numerical analyses, the consequences of the implementation of the seismic isolation system in each retrofitting case of the cable-stayed bridge led to the following conclusions:

- The base isolation retrofitting prevented the damage and failure in the tower and prevented the occurrence of damage concentration in the cable-stayed bridge. It also reduced the transmission of seismic forces from the substructure to the superstructure.
- The base isolation system at the tower base and the deck-tower connection increased the flexibility of the bridge in the longitudinal direction while the utilization of the base isolators at the end supports increased the flexibility of the bridge in the transverse direction, and, hence, minimized the longitudinal and transverse induced seismic forces, respectively.
- In both directions, the cable forces variation substantially reduced in almost all the cases except case 3. The variation of the cable forces had a significant influence on the deck stability and the reduction of the variations in the forces in the cables, which is helpful in reducing oscillation of the deck.
- The longitudinal seismic performance of the cable-stayed bridge improved in cases 1, 2, 4, and 5. In case 3, the seismic performance of the bridge only improved in the transverse direction. The base isolators at the abutments limited the longitudinal movement of the bridge, which led to an incrementation in the base shear and the base moment. Further, only cases 4 and 5 showed significant seismic improvement in the transverse direction.
- Partial seismic isolation of the bridge only led to an improvement in the seismic response of the cable-stayed bridge in one direction. In addition, the changes to the supports of the cable-stayed bridge significantly influenced its seismic behavior.
- To maximize the benefits of the isolation system for the overall enhancement of the seismic performance of the bridge in the longitudinal and transverse directions, it is necessary to utilize the isolation system along the supports and deck-tower connection of the cable-stayed bridge.

## References

[1] Ali H., Abdel-ghaffar AM. Seismic Passive Control of Cable-Stayed Bridges. Shock Vib. 1995;2(4):259–72.

[2] Ali H., Abdel-Ghaffar AM. Modeling the nonlinear seismic behavior of cable-stayed bridges with passive control bearings. Comput Struct. 1995;54(3):461–92.

[3] Ghaedi K, Ibrahim Z. Earthquake Prediction In: Zouaghi T, editor. Earthquakes—Tectonics, Hazard and Risk Mitigation. InTech; 2017 p. 205–27.

[4] Ghaedi K, Ibrahim Z, Adeli H, Javanmardi A. Invited Review: Recent developments in vibration control of building and bridge structures. J Vibroengineering. 2017;19(5):3564–80.

[5] Javanmardi A, Ibrahim Z, Ghaedi K, Khatibi H. Numerical analysis of vertical pipe damper. In: IABSE SYMPOSIUM; Engineering the Future. VANCOUVER: International Association for Bridge and Structural Engineering; 2017. p. 2974–80.

[6] Javanmardi A. Non-linear Test of Precast Subframe Subjected to Cyclic Lateral Loadings. Universiti Teknologi Malaysia; 2014.

[7] Javanmardi A, Abadi R, Marsono AK, Tap M, Ibrahim Z, Ahmad A. Correlation of Stiffness and Natural Frequency of Precast Frame System. Appl Mech Mater Vol. 2015;735:141–4.

[8] Ghaedi K, Ibrahim Zainah, Javanmardi Ahad, Hamed Khatibi MJ. Seismic Response Analysis of Fully Base Isolated Adjacent Buildings with Segregated Foundations. Adv Civ Eng [Internet]. 2017; Available from: https://www.hindawi.com/journals/ace/aip/4517940/

[9] Cunha A, Caetano E, Delgado R. Dynamic tests on large cable-stayed bridge. Bridg Eng. 2001;6(February):54–62.

[10] Ren W-X, Obata M. Elastic-Plastic Seismic Behavior of Long Span Cable-Stayed Bridges. Bridg Eng. 1999;4(December):1404–12.

[11] Ali HM, Abdel-ghaffar AM. Seismic energy dissipation for cable-stayed bridges using passive devices. Earthq Eng Struct Dyn. 1994;23(December 1991):877–93.

[12] Mozos CM, Aparicio AC. Parametric study on the dynamic response of cable stayed bridges to the sudden failure of a stay, Part I: Bending moment acting on the deck. Eng Struct [Internet]. 2010;32(10):3288–300. Available from: 10.1016/j.engstruct.2010.07.003

[13] Xi Y., Kuang JS. An energy approach for geometrically non-linear analysis of cable-stayed bridges. ICE—Struct Build. 2000;140(3):227–37.

[14] Wang PH, Yang CG. Parametric studies on cable-stayed bridges. Comput Struct. 1996;60(2):243–60.

[15] Atmaca B, Ates S. Construction stage analysis of three-dimensional cable-stayed bridges. Steel Compos Struct. 2012;12(5):413–26.

[16] Nazmy AS, Abdel-Ghaffar AM. Effects of Ground Motion Spatial Variability on The Response of Cable-Stayed Bridges. J Earthq Eng Struct Dyn [Internet]. 1992;21(1):1–20. Available from: http://www.scopus.com/inward/record.url?eid=2-s2.0-0026626765&partnerID=40&md5=a69960dc60706cb22a9e146cc5e0a953

[17] Sharabash AM, Andrawes BO. Application of shape memory alloy dampers in the seismic control of cable-stayed bridges. Eng Struct [Internet]. 2009;31(2):607–16. Available from: 10.1016/j.engstruct.2008.11.007

[18] LI H, LIU J, OU J. Investigation of seismic damage of cable-stayed bridges with different connection configuration. J Earthq Tsunami. 2009;3(3):227–47.

[19] Tuladhar R, Dilger WH. Effect of support conditions on seismic response of cable-stayed bridges. Can J Civ Eng. 1999;26(5):631–45.

[20] Iemura H, Pradono MH. Passive and semi-active seismic response control of a cable-stayed bridge. J Struct Control [Internet]. 2002;9(3):189–204. Available from: http://doi.wiley.com/10.1002/stc.12

[21] Bruno D, Greco F, Lonetti P. Dynamic impact analysis of long span cable-stayed bridges under moving loads. Eng Struct. 2008;30(4):1160–77.

[22] Camara a., Astiz M a. Pushover analysis for the seismic response prediction of cable-stayed bridges under multi-directional excitation. Eng Struct [Internet]. 2012;41:444–55. Available from: 10.1016/j.engstruct.2012.03.059

[23] Mozos CM, Aparicio a. C. Parametric study on the dynamic response of cable stayed bridges to the sudden failure of a stay, Part II: Bending moment acting on the pylons and stress on the stays. Eng Struct [Internet]. 2010;32(10):3301–12. Available from: 10.1016/j.engstruct.2010.07.003

[24] Chang C-M, Loh C-H. Seismic Response Control of Cable-Stayed Bridge Using Different Control Strategies. J Earthq Eng. 2006;10(4):481–508.

[25] He WL, Agrawal a. K. Passive and hybrid control systems for seismic protection of a benchmark cable-stayed bridge. Struct Control Heal Monit. 2007;14(1):1–26.

[26] He M, Ruiguang Hu, Chen L, Chen C, Wang J. Intelligent active control of a benchmark cable-stayed bridge. Struct Build. 2015;168(168):890–901.

[27] Wesolowsky MJ, Wilson JC. Seismic isolation of cable-stayed bridges for near-field ground motions. Earthq Eng Struct Dyn [Internet]. 2003;32(13):2107–26. Available from: 10.1002/eqe.318

[28] Dolce M, Cardone D, Palermo G. Seismic isolation of bridges using isolation systems based on flat sliding bearings. Bull Earthq Eng [Internet]. 2007;5(4):491–509. Available from: http://link.springer.com/10.1007/s10518-007-9044-3

[29] Karalar M, Padgett JE, Dicleli M. Parametric analysis of optimum isolator properties for bridges susceptible to near-fault ground motions. Eng Struct [Internet]. 2012;40:276–87. Available from: 10.1016/j.engstruct.2012.02.023

[30] Dicleli M, Mansour MY. Seismic retrofitting of highway bridges in Illinois using friction pendulum seismic isolation bearings and modeling procedures. Eng Struct. 2003;25(9):1139–56.

[31] Atmaca B, Ates S. Determination of bearing type effect on elastomeric bearing selection with SREI-CAD. Adv Comput Des. 2017;2(1):43–56.

[32] Martínez-Rodrigo MD, Filiatrault a. A case study on the application of passive control and seismic isolation techniques to cable-stayed bridges: A comparative investigation through non-linear dynamic analyses. Eng Struct [Internet]. 2015;99:232–52. Available from: http://linkinghub.elsevier.com/retrieve/pii/S0141029615003041

[33] Javanmardi A, Ibrahim Z, Ghaedi K, Jameel M, Khatibi H, Suhatril M. Seismic response characteristics of a base isolated cable-stayed bridge under moderate and strong ground motions. Arch Civ Mech Eng [Internet]. 2017;17(2):419–32. Available from: 10.1016/j.acme.2016.12.002

[34] Casciati F, Cimellaro GP, Domaneschi M. Seismic reliability of a cable-stayed bridge retrofitted with hysteretic devices. Comput Struct. 2008;86(17–18):1769–81.

[35] Park K-S, Jung H-J, Lee I-W. HYBRID CONTROL STRATEGIES FOR SEISMIC PROTECTION OF BENCHMARK CABLE-STAYED BRIDGES.

[36] Atmaca B, Yurdakul M, Ateş Ş. Nonlinear dynamic analysis of base isolated cable-stayed bridge under earthquake excitations. Soil Dyn Earthq Eng [Internet]. 2014;66:314–8. Available from: http://linkinghub.elsevier.com/retrieve/pii/S0267726114001717

[37] Filiatrault A, Tinawai R, Massicotte B. Damage to cable-stayed bridge during 1988 Saguenay earthquake. I: Pseudostatic analysis. J Struct Eng. 1993;119(5):1432–49.

[38] Filiatrault A, Tinawai R, Massicotte B. Damage to cable-stayed bridge during 1988 Saguenay earthquake. II: Dynamic analysis. J Struct Eng. 1993;119(5):1450–63.

[39] AASHTO. Guide Specifications for Seismic Isolation Design. Third Washington DC: American Association of State Highway and Transportation Officials; 2010 47 p.

[40] AASHTO. LRFD Bridge Design Specifications. Washington DC: American Association of State Highway and Transportation Officials; 2012 1661 p.

[41] Computers and Structures Inc. CSI Analysis Reference Manual. Berkeley, CA; 2015 496 p.

[42] Filiatrault A, Tinawai R, Massicotte B. Damage to cable-stayed bridge during 1988 Saguenay earthquake. I: Pseudostatic analysis. 1993;119(5):1432–49.

[43] Felber AJ, Stiemer SF. An object oriented programming approach to ambient vibration data analysis for bridges. In: Can Soc for Civ Engrg Annu Conf. Quebec City, Canada; 1992. p. 375–84.

[44] Robinson WH, Tucker AG. A lead-rubber shear damper. Bull New Zeal Natl Soc EarthquakeEngineering. 1977;10(3):151–3.

[45] Built SM. Lead rubber dissipators for the base isolation of bridge structures. University of Auckland; 1982.

[46] Park YJ, Wen YK, Ang A. Random vibration of hysteretic systems under bi-directional ground motions. Earthq Eng Struct Dyn. 1986;14(4):543–57.

[47] Dyke SJ, Caicedo JM, Turan G, Bergman L a., Hague S. Phase I Benchmark Control Problem for Seismic Response of Cable-Stayed Bridges. J Struct Eng [Internet]. 2003;129(7):857–72. Available from: http://ascelibrary.org/doi/ref/10.1061/(ASCE)0733-9445(2003)129:7(857)

